# lncRNAs Regulate Innate Immune Responses and Their Roles in Macrophage Polarization

**DOI:** 10.1155/2018/8050956

**Published:** 2018-02-11

**Authors:** Zhen Wang, Ying Zheng

**Affiliations:** ^1^Department of Metabolism and Endocrinology, The Second Xiangya Hospital, Central South University, Changsha, Hunan 410011, China; ^2^Key Laboratory of Diabetes Immunology, Central South University, Ministry of Education, National Clinical Research Center for Metabolic Diseases, Changsha, Hunan 410011, China; ^3^Center for Medical Research, The Second Xiangya Hospital, Central South University, Changsha, Hunan 410011, China

## Abstract

The innate immune system is the first line of defense against microbial pathogens. The activated innate immune system plays important roles in eliciting antimicrobial defenses. Despite the benefits of innate immune responses, excessive inflammation will cause host damage. Thus, tight regulation of these processes is required for the maintenance of immune homeostasis. Recently, a new class of long noncoding RNAs (lncRNAs) has emerged as important regulators in many physiological and pathological processes. Dysregulated lncRNAs have been found to be associated with excessive or uncontrolled inflammation. In this brief review, we summarize the roles of functional lncRNAs in regulating innate immune responses. We also discuss the roles of lncRNAs in macrophage polarization, an important molecular event in the innate immune responses.

## 1. Introduction

The first line of host defense against microbial pathogens is the innate immune system that is primarily composed of innate immune molecules and myeloid-derived immune cells, including monocytes, macrophages, dendritic cells (DCs), and granulocytes [1]. When infection occurs, the surface or intracellular pattern recognition receptors (PPRs) of innate immune cells, such as Toll-like receptors (TLRs), Nod-like receptors (NLRs), and the receptor for advanced glycation end products (RAGE), recognize pathogen-associated molecular patterns (PAMPs) and damage-associated molecular patterns (DAMPs) [2, 3]. The engagement of PAMPs or DAMPs with specific receptors initiates a cascade of signaling events, subsequently leading to the production of proinflammatory cytokines, chemokines, reactive oxygen and nitrogen species, and antimicrobial peptides, as well as enhanced phagocytic activity and the rapid removal of infection [2, 3]. In addition, the activation of innate immune system favors the subsequent activation of specific adaptive immunity [4]. Despite the benefits of innate immune system, it can be a double-edged sword as excessive inflammation will cause host damage [5]. Therefore, it is important for us to understand the regulatory mechanisms that control the initiation, magnitude, and resolution of the inflammatory process.

In recent years, a new class of noncoding RNAs, long noncoding RNAs (lncRNAs), has emerged as novel players in the regulation of gene expression [6, 7]. Accumulating studies have shown the importance of lncRNAs in the regulation of immune and inflammatory responses [8–11]. Dysregulation of lncRNAs has also been demonstrated to contribute to the aberrant immune responses and inflammatory diseases [12]. In this review, we will summarize the regulatory roles of lncRNAs in innate immune response. We also discuss the effects of lncRNAs on macrophage polarization, an important molecular event that has clear impact on inflammation, wound repair, and tumor progression.

## 2. lncRNAs: A Novel Class of Noncoding RNAs Regulating Gene Expression

**Figure 1:**
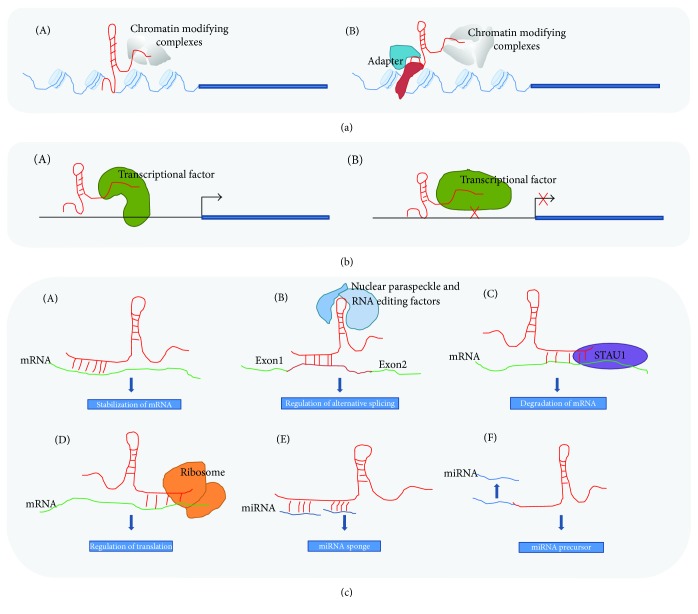
The diverse function mechanisms of lncRNAs. (a) lncRNAs recruit chromatin-modifying complexes onto specific chromosomal loci, subsequently regulating gene expression in *cis* (A) and in *trans* (B). (b) lncRNAs interact with transcription factors and influence the localization and activity of the transcription factors, subsequently regulating gene expression. (A) Transcriptional activation. lncRNAs recruit specific transcriptional factors onto specific chromosomal loci, thus facilitating gene transcription. (B) Transcriptional repression. lncRNAs bind with transcription factors and prevent the binding of the transcription factors with the promoter, subsequently mediating gene silencing. (c) lncRNAs in posttranscriptional regulation. (A) Stabilization of mRNA. By base pairing with mRNA, lncRNAs can increase stabilization of the target mRNA. (B) Regulation of alternative splicing. By base pairing with mRNA, lncRNAs may participate in the formation and maintenance of nuclear paraspeckles, which can facilitate alternative splicing events of nascent transcripts. (C) Degradation of mRNA. Through base pairing between mRNA and lncRNAs, staufen double-stranded RNA-binding protein 1- (STAU1-) mediated mRNA decay is induced. (D) Regulation of translation. By base pairing with mRNA and interacting with ribosomal proteins, lncRNAs can target mRNA to the ribosomes and affect the translation. (E) miRNA sponge. lncRNAs function as molecular “sponges” for miRNAs and regulate the expression of the miRNA target genes. (F) miRNA precursor. lncRNAs can serve as a source of miRNAs after processing. lncRNAs are shown in red, whereas miRNAs are in blue.

lncRNAs are a large group of non-protein coding transcripts, which are more than 200 nucleotides in length but lack protein-coding potential [7]. Based on their genomic localization and position relative to protein-coding gene, lncRNAs are classified into the long intergenic ncRNA (transcribed between known protein-coding genes, lincRNA), intronic lncRNA (transcribed within introns of a protein-coding gene), sense lncRNA (transcribed from a gene but lack the ability of producing protein), natural antisense transcription (transcribed across the exons of a protein-coding gene from the opposite direction, NAT), and bidirectional transcription (transcribed in opposite directions in relation to the promoter of a protein-coding gene) [13]. Most lncRNAs are transcribed by RNA polymerase II and are spliced and modified with a 5′ cap and a poly-A tail, all of which make them largely indistinguishable from mRNAs [13]. In contrast to mRNAs, lncRNAs lack protein-coding capacity. The mechanisms by which lncRNAs regulate gene expression are still incompletely clear. Emerging evidence has suggested that lncRNAs can control gene expression at the levels of epigenetic control, transcription, RNA processing, and translation (Figure 1) [13, 14]. Many lncRNAs have been found to be significantly enriched in the chromatin fraction, and a common function of these lncRNAs is to recruit chromatin-modifying complexes, including the Polycomb group (PcG) or Trithorax group (TrxG), to create a repressive chromatin state or an active chromatin state and affect gene expression either in *cis* or in *trans* to distant target genes (Figure 1(a)) [13–15]. Alternatively, some lncRNAs can form RNA-protein complexes with transcription factors and influence the localization and activity of the transcription factors that they bind, subsequently regulating gene expression (Figure 1(b)) [13–15]. Aside from the aforementioned diverse mechanisms, lncRNAs also participate in the control of the posttranscriptional events of mRNAs, including the maintenance of RNA stabilization, RNA splicing, translation or degradation, and miRNA generation or regulation (Figure 1(c)) [13–15]. Recently, some lncRNAs have been found to encode small peptides as their functional elements [16]. A large number of studies have revealed that lncRNAs play vital roles in the transcriptional and posttranscriptional regulation of gene expression in a variety of biological and pathogenic processes, such as X chromosome inactivation, genomic imprinting, stem cell pluripotency and development, tumor growth and metastasis, and immune responses [13, 17–19]. Accumulating evidence also supports important roles of lncRNAs in regulating the innate immune system [9, 10, 12, 20].

## 3. lncRNAs in Innate Immune Responses

Recently, the functional roles of lncRNAs in innate immune responses have emerged to call for our attention. lncRNA expression has been found to be changed in the process of innate immune response. Changed lncRNAs play important roles in regulating innate immune responses (Table 1). And dysregulated lncRNAs are associated with the onset and development of inflammatory diseases.

### 3.1. lincRNA-Cox2

lincRNA-Cox2 was defined as it is located ~51 kb upstream of the protein-coding gene Cox2, a crucial inflammatory mediator that can be induced by the transcription factor NF-*κ*B upon TLR4 stimulation [21]. lincRNA-Cox2 was found to be markedly upregulated by over 1000-fold after TLR4 stimulation in CD11C^+^ bone-marrow-derived dendritic cells (BMDCs), but it was just weakly increased following the stimulation of TLR3 ligands. And later, lincRNA-Cox2 was also demonstrated to be induced in macrophages stimulated with Pam3CSK4 and R848, which are the agonists for TLR2/6 and TLR7/8, respectively [22]. Induction of lincRNA-Cox2 by TLR ligands is dependent on the TLR signaling adaptor protein MyD88 and on the activation of NF-*κ*B. Furthermore, lincRNA-Cox2 was proved to control the activation and inactivation of different groups of inflammatory genes, and thus, it is an important regulator of immune response. In resting macrophages, knockdown of lincRNA-Cox2 leads to increased expression of more than 700 genes, including a number of chemokines (CCL5, CX3CL1), chemokine receptors (CCRl), and interferon-stimulated genes (ISGs) (IRF7, OAS1a, OaS1l, OAS2, IFI204, and ISG15). In Pam3CSK4-stimulated macrophages, silencing of lincRNA-Cox2 results in attenuated expression of 713 genes, including TLR1, IL-6, and IL-23a. The regulatory effect of lincRNA-Cox2 on immune gene expression was demonstrated to associate with hnRNP-A/B and A2/B1. lincRNA-Cox2 can interact with hnRNP-A/B and A2/B1 to form a complex, ultimately repressing the transcription of immune genes. Similarly, lincRNA-Cox2 was also observed to have impact on the reprogramming of the gene expression profile in intestinal epithelial cells (IECs) with the stimulation of TNF-*α* [23]. lincRNA-Cox2 is drastically increased in TNF-*α*-treated IECs. Upregulated lincRNA-Cox2 represses the transcription of IL-12b in response to TNF-*α* stimulation, and it mediates this effect by its recruitment of the Mi-2/nucleosome remodeling and deacetylase (Mi-2/NuRD) repressor complex to the IL-12b promoter region. Recently, it was evident that lincRNA-Cox2 is essential for the transcription of NF-*κ*B-mediated late-primary inflammatory response genes [24]. Specifically, after LPS stimulation, lincRNA-Cox2 is assembled into the SWI/SNF complex in macrophages, and then the lincRNA-Cox2/SWI/SNF complex mediates the assembly of NF-*κ*B subunits into the SWI/SNF complex, subsequently mediating SWI/SNF-associated chromatin remodeling and transcriptional activation of the late-primary inflammatory response genes.

### 3.2. PACER

p50-associated COX-2 extragenic RNA (PACER) is another COX2-associated lncRNA that is transcribed from the antisense orientation upstream of Cox2 transcription start site [25]. This antisense lncRNA is a positive regulator of COX-2 expression in human mammary epithelial cells and in monocyte-derived macrophage-like cells before and after LPS stimulation. It was observed that PACER is modulated by the chromatin-boundary/insulator factor CCCTC-binding factor (CTCF)/cohesin complex, which creates a permissive chromatin environment in the upstream promoter region of COX-2 to promote PACER transcription. In turn, upregulated PACER interacts with the repressive NF-*κ*B subunit p50 and enables it divorced from the COX-2 promoter, thereby facilitating the formation of the active NF-*κ*B p65/p50 dimers. Eventually, these events favor the recruitment of the p300 histone acetyltransferase and RNA polymerase II (RNAPII) preinitiation complexes in the promoter region of Cox2 to promote COX-2 transcription.

### 3.3. Lethe

Lethe was the first confirmed functional pseudogene lncRNA in the mammalian genome [26]. It is 697 bp long unspliced lncRNA and located approximately 500 bp downstream of Gmeb1 and 8 kb upstream of Ythdf2 on the minus strand on chromosome 4. Lethe is selectively induced by proinflammatory cytokine TNF-*α* and IL-1*β* and the anti-inflammatory agent, dexamethasone, in mouse embryonic fibroblast (MEF) cells. The induction of lethe is dependent on NF-*κ*B activation. On the other hand, lethe regulates NF-*κ*B signaling in the negative feedback way. Specifically, lethe binds to the active NF-*κ*B subunit p65 (RelA) and prevents it from binding to the promoters of target genes, thus reducing the production of a number of inflammatory proteins, such as IL-6, IL-8, and superoxide dismutase 2 (SOD2). Additionally, lethe expression was found to decrease with aging. As is well known, aging is strongly associated with NF-*κ*B activation [27], and thus, there is reason to believe that the age-dependent downregulation of lethe is likely to be one cause for increasing NF-*κ*B activity during aging. Therefore, lethe appears to function as a novel negative modulator of NF-*κ*B to help control inflammatory responses.

### 3.4. THRIL

TNF-*α* and heterogenous nuclear ribonucleoprotein L- (hnRNPL-) related immunoregulatory LincRNA (THRIL), also known as linc1992, was first identified in the human monocyte cell line THP1-differenitated macrophages following stimulation with Pam3CSK4 [28]. THRIL is expressed in many human tissues. Knockdown of THRIL leads to a strong reduction of TNF-*α* and IL-6 secretion. And THRIL is essential for the induction of TNF*α* expression. Actually, THRIL interacts with hnRNPL to establish a functional THRIL-hnRNPL complex, consequently regulating TNF-*α* transcription by binding to its promoter. The transcriptome analysis by RNA sequencing in THRIL-deficient THP-1-derived macrophages with the stimulation of Pam3CSK4 indicated that THRIL has broad effects on innate immunity-associated gene expression. In addition, THRIL expression was found to correlate with the severity of Kawasaki disease, an acute inflammatory disease of childhood, which also hinted the important roles of THRIL in inflammatory responses and inflammatory diseases.

### 3.5. NEAT1

Nuclear-enriched abundant transcript 1 (NEAT1) is a nuclear noncoding RNA that is essential for the formation of nuclear body paraspeckles [29]. NEAT1 can be induced by influenza virus, or herpes simplex virus (HSV) infection [30]. Its expression was also reported to increase after the stimulation of the TLR3 ligand poly I:C, a double-stranded RNA (dsRNA) [30]. Upon stimulation, increased NEAT1 promotes the production of antiviral cytokines, such as IL-8. The details of NEAT1's antiviral function are involved with splicing factor proline/glutamine rich (SFPQ), a NEAT1-binding paraspeckle protein that represses IL-8 transcription. Induced NEAT1 can relocate SFPQ from the IL-8 promoter to the paraspeckles, consequently resulting in the activation of IL-8 transcription. Additionally, NEAT1 is changed by HIV-1 infection, and knockdown of NEAT1 can enhance virus production by increasing nucleus-to-cytoplasm export of HIV-1 mRNA [31].

### 3.6. AS-IL-1*α*

Antisense-interleukin 1*α* (AS-IL-1*α*) is the first confirmed functional innate immune NAT that is complementary to IL-1*α* [32]. Resting macrophages have a lower level of AS-IL-1*α*. However, when the cells are infected with *Listeria monocytogenes* or stimulated with TLR ligands such as Pam_3_CSK_4_, LPS, and Poly I:C, AS-IL-1*α* is strongly induced. AS-IL-1*α* functions as an important player in regulating IL-1*α* transcription. It can be located in the nucleus and favors the recruitment of RNAPII to the IL-1*α* promoter, thus controlling the transcriptional activation of IL-1*α*.

### 3.7. Lnc-IL7R

A newly reported immune-related lncRNA is Lnc-IL7R. It overlaps with the 3′ untranslated region (3′ UTR) of the human interleukin-7 receptor *α*-subunit gene. This lncRNA is remarkably increased in THP-1 cells after being exposed to LPS [33]. Upregulated Lnc-IL7R in turn diminishes LPS-mediated inflammatory responses, characterized by the reduction of LPS-induced E-selectin, VCAM-1, IL-6, and IL-8 expression. Lnc-IL7R appears to suppress the expression of E-selectin and VCAM-1 by regulating trimethylation of histone H3 at lysine 27 (H3K27me3) and increasing its level at the promoters of the two genes. But the process of Lnc-IL7R regulating IL-8 expression is not involved with H3K27me3, and the exact mechanism needs to be deeply studied in the future. Recently, it was shown that in oral squamous cell carcinoma (OSCC) cells, Lnc-IL7R expression is upregulated in response to poly I:C and/or combined chemotherapy, accompanied with decreased TLR3 expression [34]. And knockdown of TLR3 can upregulate the Lnc-IL7R expression in OSCC cells. Thus, these data revealed that poly I:C and/or combined chemotherapy enhances the expression of Lnc-IL7R via TLR3. Furthermore, upregulated Lnc-IL7R is associated with chemotherapy resistance in the carcinogenesis of OSCC.

### 3.8. Lnc-DC

By transcriptome microarray analysis and RNA sequencing (RNA-seq), Wang et al. identified a modestly conserved intergenic lncRNA, namely Lnc-DC, which is exclusively expressed in human conventional DCs [35]. Wang et al. showed that the specific high expression of Lnc-DC in human DCs is ascribed to accessible chromatin structure, active histone modifications, and binding of the transcription factor PU.1 at the promoter region of Lnc-DC. Lnc-DC exerts important roles in human DC differentiation from monocytes and DC functions. For example, knockdown of Lnc-DC leads to an obvious change of many DC function-related genes, including downregulated CD40, CD80, CD86, and HLA-DR that are essential for T cell activation and upregulated CD14 that is the exclusive marker for monocytes. Functionally, knockdown of Lnc-DC can cause an impaired antigen uptake by Mo-DC and also compromise their capability to induce the proliferation of allogeneic CD4^+^ T cells. In addition, knockdown of Lnc-DC can suppress the induction of IL-12 by LPS stimulation. The regulatory effect of Lnc-DC on DCs involves STAT3 signaling, which is critical for DC development and function. In fact, Lnc-DC binds directly with the C terminus of STAT3, thus promoting STAT3 phosphorylation on tyrosine-705 by preventing STAT3 dephosphorylation. Preeclampsia patients were found to have enhanced Lnc-DC expression and STAT3 phosphorylation in the deciduas, along with increased proportion of decidual mature DCs and a bias of CD4^+^ T cell differentiation into Th1 phenotype [36]. And it is therefore speculated that upregulated Lnc-DC promotes the overmaturation of DCs and improves their ability to induce CD4^+^ T cells to develop into Th1 cells, therefore mediating inflammatory responses and contributing to the pathogenesis of preeclampsia. Moreover, it was recently reported that plasma levels of Lnc-DC are significantly decreased in systemic lupus erythematosus (SLE) patients, and the levels of Lnc-DC are considerably higher in SLE with nephritis, when compared with whom without nephritis [37]. Thus, Lnc-DC in plasma could be a potential biomarker for SLE. Of note, there are some questions about the murine orthologue of Lnc-DC, because this gene was previously identified as Wdnm1-like, and its transcript can be translated into the Wdnm1-like protein in most mammals but with humans as an exception which is possibly unique [38, 39]. So, whether the function of this gene in mouse is attributed to the Wdnm1-like transcript or the Wdnm1-like protein needs for further investigation.

### 3.9. HOTAIRM1

HOX antisense intergenic RNA myeloid 1 (HOTAIRM1) is an antisense lncRNA, which was identified to associate with the granulocyte maturation [40]. It is specifically expressed in myeloid cells. During granulocyte differentiation driven by retinoic acid (RA), HOTAIRM1 appears to be significantly upregulated. Knockdown of HOTAIRM1 can partially counteract RA-induced expression of HOXA1 and HOXA4 during the RA-induced granulocyte differentiation of NB4 cells and selectively attenuated induction of CD11b and CD18 expression, two hallmarks for granulocyte differentiation.

### 3.10. IL-1*β*-eRNA and IL1*β*-RBT46

Ilott et al. identified 76 enhancer RNAs (eRNAs), 40 canonical lncRNAs, 65 antisense lncRNAs, and 35 regions of bidirectional transcription (RBT) differentially expressed in human monocytes after the stimulation of LPS [41]. Among them, an eRNA (IL-1*β*-eRNA) and a RBT (IL1*β*-RBT46) that surround the IL-1*β* locus, were found to play important roles in innate immune responses. Actually, knockdown of IL-1*β*-eRNA or IL1*β*-RBT46 selectively attenuates LPS-induced production of the proinflammatory factors, such as IL-1*β* and CXCL8.

### 3.11. FIRRE

Functional intergenic repeating RNA element (FIRRE) is a newly discovered lncRNA that can anchor the inactive X chromosome through maintaining H3K27me3 methylation [42]. It functions as a nuclear-organization factor and has an impact on the higher-order nuclear architecture across chromosomes through interacting with hnRNPU [43]. It is a conserved lncRNA between humans and mice. Recently, Lu et al. found that FIRRE can be induced in the human macrophages and intestinal epithelial cells with the stimulation of LPS [44]. And its expression is controlled by NF-*κ*B signaling. Moreover, FIRRE can positively regulate the expression of several inflammatory genes at the posttranscriptional level through its interaction with hnRNPU.

## 4. The Roles of lncRNAs in Macrophage Polarization

Macrophages exert extremely important roles in the inflammatory responses because of their ability to detect and engulf the pathogens. However, depending on the microenvironment, macrophages undergo specific differentiation with two distinct functional phenotypes, proinflammatory macrophages (M1 macrophages) and alternatively activated, anti-inflammatory macrophages (M2 macrophages) [45–47]. The macrophages that produce proinflammatory cytokines and inducible NO synthase (iNOS) are known as proinflammatory macrophages (also called classically activated macrophages), and anti-inflammatory macrophages are characterized by the release of anti-inflammatory cytokines, increasing arginase-1 (Arg-1) activity, and high levels of CD206, CD163, CCL17, and CCL22 [45–49]. The proinflammatory macrophages are induced mainly by Th-1 cytokines and TLR ligands. On the contrary, the stimulation with Th-2 cytokines will enable macrophages to develop into the anti-inflammatory macrophages [45, 46, 49]. Macrophage polarization associates with the pathophysiologic states in humans and it plays a crucial role in many diseases, including infection, atherosclerosis, cancer, and so on [50, 51]. Thus, it is important for us to explore the regulatory mechanisms of dynamic transition between different macrophage phenotypes.

Recently, Huang and his colleagues identified differentially expressed lncRNAs in polarized macrophages. In their study, lncRNA expression profiles were detected in human monocyte-derived macrophages with the treatment of IFN-*γ* + LPS or IL-4. As we discussed above, macrophages can be polarized to proinflammatory macrophages by the stimulation of IFN-*γ* + LPS, whereas anti-inflammatory macrophages are polarized by the exposure to Th2 cytokines such as IL-4. Compared with primary monocyte-derived macrophages, 9343 lncRNAs were found to be deregulated in proinflammatory macrophages, while 4592 lncRNAs were deregulated in anti-inflammatory macrophages. They found that lncRNA TCONS_00019715 has higher expression in proinflammatory macrophages than that in anti-inflammatory macrophages. When proinflammatory macrophages convert to anti-inflammatory macrophages, TCONS_00019715 expression decreases. However, it increases when anti-inflammatory macrophages convert to proinflammatory phenotype. Knockdown of TCONS_00019715 diminishes the expression of proinflammatory macrophage markers and increases the expression of anti-inflammatory markers. TCONS_00019715 promotes macrophage transition to proinflammatory macrophages by downregulating PAK1, an important regulator of cytoskeletal remodeling and cell motility in mononuclear phagocytic system [52]. These data revealed important roles of lncRNA TCONS_00019715 in regulating macrophage polarization. Another example is lncRNA E330013P06, which was found to increase in macrophages from db/db and diet-induced insulin-resistant T2D mice and monocytes from T2D humans [53]. E330013P06 overexpression can regulate proinflammatory gene expression and foam cell formation in macrophages. Furthermore as discussed above, lncRNAs, including lincRNA Cox2, PACER, and Lethe, can regulate many inflammatory genes and inflammatory signaling pathways, which are involved with macrophage polarization [24, 25, 27]. Nevertheless, the roles of other functional lncRNAs in regulating macrophage polarization are needed for further investigation. Many molecules and signaling pathways are involved in macrophage polarization and its functions [54–58], and lncRNAs may participate in macrophage polarization by regulating these molecules and signaling pathways.

## 5. Conclusions

In recent years, a series of studies in the immune system have provided us a lot of evidence that lncRNAs have a vital role in regulating inflammatory responses. Dysregulated lncRNAs were found to be associated with excessive or uncontrolled inflammation condition. Considering the important effects of lncRNAs on innate immune responses, it is believed that lncRNAs may be an effective strategy for controlling excessive inflammation and maintaining immune homeostasis. Despite identification of the roles of some specific lncRNAs in regulating innate immune system, further investigation of more lncRNAs will likely shed light on their biological functions and their association with innate immune responses.

## Figures and Tables

**Table 1:** lncRNAs regulate the innate immune responses.

lncRNAs	Description of evidence	References
lincRNA-Cox2	lincRNA-Cox2 regulates the expression of inflammatory genes by binding with hnRNP-A/B and A2/B1. It also can interact with SWI/SNF complex in macrophages, to create lincRNA-Cox2/SWI/SNF complex, subsequently mediating SWI/SNF-associated chromatin remodeling and transcriptional activation of the late-primary inflammatory response genes. lincRNA-Cox2 represses the transcription of IL-12b in response to TNF-*α* stimulation.	[21–24]
PACER	PACER interacts with the repressive NF-*κ*B subunit p50, enables it divorced from the COX-2 promoter, and favors the recruitment of the p300 histone acetyltransferase and RNA polymerase II (RNAPII) preinitiation complexes in the promoter region of Cox2 to promote COX-2 transcription.	[25]
Lethe	Lethe binds to the active NF-*κ*B subunit p65 (RelA) and prevents it from binding to the promoters of target genes, thus reducing the production of inflammatory proteins, such as IL-6, IL-8, and superoxide dismutase 2 (SOD2).	[26]
THRIL	THRIL interacts with hnRNPL to establish a functional THRIL-hnRNPL complex, consequently regulating TNF-*α* transcription by binding to its promoter.	[28]
NEAT1	NEAT1 binds to SFPQ, relocating it from the IL-8 promoter to the paraspeckles and resulting in the activation of IL-8 transcription; knockdown of NEAT1 can enhance virus production by increasing nucleus-to-cytoplasm export of HIV-1 mRNA.	[29–31]
AS-IL1*α*	AS-IL1*α* favors the recruitment of RNAPII to the IL-1*α* promoter, thus controlling the transcriptional activation of IL-1*α*.	[32]
Lnc-IL7R	Lnc-IL7R regulates trimethylation of histone H3 at lysine 27 (H3K27me3) and increases its level at the promoters of E-selectin and VCAM-1, suppressing the expression of the two genes.	[33]
Lnc-DC	Lnc-DC promotes phosphorylation and activation of STAT3, a transcription essential for DC differentiation, by blocking its dephosphorylation by SHP1.	[35]
HOTAIRM1	HOTAIRM1 regulates RA-induced expression of HOXA1 and HOXA4 during the RA-induced granulocyte differentiation of NB4 cells and promotes induction of CD11b and CD18 expression, two hallmarks for granulocyte differentiation.	[40]
IL-1*β*-eRNA and IL1*β*-RBT46	IL-1*β*-eRNA or IL1*β*-RBT46 regulates LPS-induced production of the proinflammatory factors, such as IL-1*β* and CXCL8.	[41]
FIRRE	FIRRE can positively regulate the expression of several inflammatory genes at the posttranscriptional level through its interaction with hnRNPU.	[44]
